# Host Genetic Variants Potentially Associated With SARS-CoV-2: A Multi-Population Analysis

**DOI:** 10.3389/fgene.2020.578523

**Published:** 2020-10-02

**Authors:** Maria K. Smatti, Yasser A. Al-Sarraj, Omar Albagha, Hadi M. Yassine

**Affiliations:** ^1^College of Health and Life Sciences, Hamad Bin Khalifa University, Doha, Qatar; ^2^Biomedical Research Center, Qatar University, Doha, Qatar; ^3^Centre for Genomic and Experimental Medicine, Institute of Genetics and Molecular Medicine, University of Edinburgh, Edinburgh, United Kingdom

**Keywords:** COVID-19, SARS-CoV-2, host genetics, susceptibility, SNPs

## Abstract

**Background:**

Clinical outcomes of coronavirus disease 2019 (COVID-19), caused by the severe acute respiratory syndrome coronavirus 2 (SARS-CoV-2) showed enormous inter-individual and inter-population differences, possibly due to host genetics differences. Earlier studies identified single nucleotide polymorphisms (SNPs) associated with SARS-CoV-1 in Eastern Asian (EAS) populations. In this report, we aimed at exploring the frequency of a set of genetic polymorphisms that could affect SARS-CoV-2 susceptibility or severity, including those that were previously associated with SARS-CoV-1.

**Methods:**

We extracted the list of SNPs that could potentially modulate SARS-CoV-2 from the genome wide association studies (GWAS) on SARS-CoV-1 and other viruses. We also collected the expression data of these SNPs from the expression quantitative trait loci (eQTLs) databases. Sequences from Qatar Genome Programme (QGP, *n* = 6,054) and 1000Genome project were used to calculate and compare allelic frequencies (AF).

**Results:**

A total of 74 SNPs, located in 10 genes: *ICAM3*, *IFN*-γ, *CCL2*, *CCL5*, *AHSG, MBL*, *Furin*, *TMPRSS2*, *IL4*, and *CD209* promoter, were identified. Analysis of Qatari genomes revealed significantly lower AF of risk variants linked to SARS-CoV-1 severity (*CCL2*, *MBL*, *CCL5*, *AHSG*, and *IL4*) compared to that of 1000Genome and/or the EAS population (up to 25-fold change). Conversely, SNPs in *TMPRSS2*, *IFN*-γ, *ICAM3*, and *Furin* were more common among Qataris (average 2-fold change). Inter-population analysis showed that the distribution of risk alleles among Europeans differs substantially from Africans and EASs. Remarkably, Africans seem to carry extremely lower frequencies of SARS-CoV-1 susceptibility alleles, reaching to 32-fold decrease compared to other populations.

**Conclusion:**

Multiple genetic variants, which could potentially modulate SARS-CoV-2 infection, are significantly variable between populations, with the lowest frequency observed among Africans. Our results highlight the importance of exploring population genetics to understand and predict COVID-19 outcomes. Indeed, further studies are needed to validate these findings as well as to identify new genetic determinants linked to SARS-CoV-2.

## Introduction

Viruses have been replicating in vertebrates for more than 450 million years (Aiewsakun and Katzourakis, 2017). This host-pathogen interaction has exerted a selective pressure over time and affected specific allelic frequencies of certain populations to favor a particular genetic variant. The frequent outbreaks of coronaviruses in China (SARS-CoV-1 in 2003, and the current SARS-CoV-2) raised the possibility that Asians have unique genetic factors that influence their susceptibility to coronaviruses (Chen et al., 2020). In addition, the large variation in coronavirus disease 2019 (COVID-19) clinical manifestation has raised multiple questions on the underlying factors, including host genetics. Moreover, COVID-19 mortality rates were considerably variable between different regions, ranging from 0.06% (Singapore) to 15% (the United Kingdom) (Statistica, 2020). Interestingly, although Qatar has the world’s highest COVID-19 infection rate per million people (38,714 cases/million), it is one of the countries reporting the lowest severity (1% ICU cases) and mortality rates (0.16%) (Our World in Data, 2020). Although age, health condition, disease management, and health systems contribute to different disease outcomes, there is a strong indication that vulnerability to COVID-19 is influenced by host genetic architecture (Tanigawa and Rivas, 2020).

In an effort to explore the susceptibility to SARS-CoV-1 in Chinese populations, multiple GWASs were conducted. Despite the limitation in the number of SARS-CoV-1 infected individuals, few reports identified significant associations. For instance, a variant (rs2248690) in the α-2-Heremans-Schmid Glycoprotein (*AHSG*) gene, which is required for macrophage deactivation, was found to affect AHSG level in the blood (Zhu et al., 2011). Carriers of the T allele had lower AHSG serum concentration and increased risk of SARS illness (Zhu et al., 2011). Low levels of the Mannose-binding lectin (MBL), another key molecule in innate immunity, caused by a missense variant (rs1800450), was also linked to increased SARS-CoV-1 susceptibility (Tu et al., 2015). Similarly, a functional polymorphism of the chemokine (C–C motif) ligand 2 (*CCL2*) gene (rs1024611) was associated with an increased risk of SARS-CoV-1 infection (Tu et al., 2015). CCL2 belongs to the chemokines family, and plays a vital role in immune cells trafficking during SARS-CoV-1 infection (Law et al., 2005). In addition, a variant (rs2280788) within another chemokine encoding gene, *CCL5*, has been found to associate with SARS susceptibility, hospitalization, and risk of death (Ng et al., 2007). Other variants in cytokines encoding genes have also been linked to SARS outcomes. Specifically, a polymorphism in the interferon *(IFN)*-γ gene (rs2430561, A), which is essential in driving T helper cell type 1 (Th1), monocytes, and macrophages responses, showed a dose-dependent increase in the susceptibility to SARS-CoV-1 (Chong et al., 2006). IL4, on the other hand, promotes and stimulates both T-cell and B-cell differentiation, and balances Th1 and Th2 responses, therefore, directly affects infection outcomes (Choi and Reiser, 1998). A meta-analysis by Patarčić et al. (2015) reported on the association of interleukin 4 (*IL4)* polymorphism (rs2070874) and multiple respiratory infections, including SARS-CoV-1. Another key T-cell activation molecule is the intercellular adhesion molecule-3 (ICAM3). SARS-CoV-1 patients carrying a genetic polymorphism in the *ICAM3* gene (rs2304237) showed higher lactate dehydrogenase (LDH) level, lower WBC count, and thus, poorer prognosis (Chan et al., 2007). Similarly, a SNP (rs4804803) located in the *CD209*, or the dendritic cell–specific ICAM-3–grabbing nonintegrin (*DC-SIGN*) gene promoter, was associated with high LDH levels in SARS-CoV-1 patients (Chan et al., 2010). This gene encodes for an important C-type lectin that acts as a pathogen receptor. Previous studies demonstrated that CD209 interacts with the spike (S)–protein of SARS-CoV-1 and enhances spike (S)-pseudotyped SARS-CoV-1 infection in susceptible cells (Marzi et al., 2004). It was found that SARS-CoV-1 patients who carry -336A > G variant have a 60% chance of having a poorer prognosis (Chan et al., 2010).

Recent studies indicated that SARS-CoV-2 spread depends on the transmembrane serine protease 2 (TMPRSS2) for virus entry (Hoffmann et al., 2020b). Importantly, SARS-CoV-2 is characterized by the acquisition of a S1/S2 multibasic cleavage site, and therefore, other proteases, including Furin and cathepsin B/L could substitute TMPRSS2 (Hoffmann et al., 2020a,b). Considering that influenza virus entry also utilizes TMPRSS2 for the cleavage of viral hemagglutinin (HA) protein, the genetic association of *TMPRSS2* variants and influenza infection was previously investigated. Variants within *TMPRSS2* (rs2070788, rs383510) were found to increase TMPRSS2 expression and significantly correlate with influenza A(H1N1)pdm09 and A(H7N9) susceptibility and severity (Cheng et al., 2015). On the other hand, there was no GWAS on the association between *cathepsin B/L* (*CTSB/CTSL)* variants and viral infections, and only one GWAS identified a variant (rs4932178, T) in *Furin* promoter that was linked to *Furin* upregulation in hepatitis B patients (Lei et al., 2009). Given the similarity between the novel SARS-CoV-2 and SARS-CoV-1, as well as the involvement of different proteases in the SARS-CoV-2 pathogenesis, this study investigates and compares the frequency of the above-mentioned variants among the Qatari as well as other populations.

## Materials and Methods

### Study Subjects

The present study included a subpopulation (*n* = 6,218) from a cohort of 10,694 participants in Qatar Biobank. A detailed demographic characterization of this cohort has been previously documented (Al Thani et al., 2019). Briefly, Qatar Biobank enrolls adults (age ≥ 18 years), following certain inclusion/exclusion criteria to obtain a representative sample of the permanent heterogeneous population that resides in Qatar. All subjects are Qatari nationals or long-term residents (≥15 years living in Qatar). Qatari individuals represent 85% of the total number of QBB participants, while long-term residents (LTR)-Arabs and LTR-Non-Arabs represent 12% and 3%, respectively. The mean age of enrolled subjects was 40.5 years (SD ± 12).

### Genomic Data

Whole-genome sequences of 6,218 Qatari nationals or long-term residents who had previously participated in Qatar Genome Programme (QGP) were obtained. Sequencing read data were previously generated by Illumina HiSeq X Ten1 sequencers and converted from the native BCL format to paired-end FASTQ2 format using bcl2fastq3. The quality of the raw data was assessed using fastqc4. Data passing quality control was then aligned to the reference genome sequence [build GRCh37 (hs37d5)5] using the bwa-kit6 aligner [v7.12]. Variant calling was performed using GATK7 haplotype caller [v3.3], and annotation of the resulting VCF8 was performed using snpeff9 [v4.1b] and the following databases: dbsnp10 v138 and dbNSFP11 v2.9. The genetic variant data was then converted to PLINK file format using PLINK-1.9 (Purcell et al., 2007). Standardized quality-assurance and quality control (QA/QC) methods were followed to generate high quality and confidence on both SNPs and sample levels, as previously described (Albagha et al., 2011). Briefly, variants with genotype call rate < 90%, Hardy-Weinberg *p*-value < 1 × 10^–6^ were removed. Samples with excess heterozygosity (*n* = 8), duplicates (*n* = 10), call rate < 95% (*n* = 1), gender ambiguity (*n* = 65), and population outlier (*n* = 87) were removed. The final file used for calculating allele frequency contained 6,047 subjects.

### SNPs Data Extraction

Data on SNPs previously linked to SARS-CoV-1 susceptibility were collected from previous GWAS (Chong et al., 2006; Chan et al., 2007, 2010; Ng et al., 2007; Lei et al., 2009; Zhu et al., 2011; Cheng et al., 2015; Patarčić et al., 2015; Tu et al., 2015). SNPs associated with susceptibility to other viral infections located in genes significant for SARS disease were also included in the analysis. Only SNPs that were reported to have significant associations with viral infections (*p*-value < 0.05) were included. For the same set of genes, quantitative trait loci (eQTLs) were retrieved from eQTLs datasets^1^ ^,2^. Only SNPs showing robust association with gene expression (association *p*-value < 1 × 10^–8^) were extracted. Subsequently, the effect of SNPs on gene expression in whole blood was obtained from the genotype-tissue expression (GTEx) database^3^.

### Data Analysis

PLINK v1.9 was used to prune eQTL SNPs into a list of independent variants (Purcell et al., 2007). SNPs within a window of 25 kb and with linkage disequilibrium *r*^2^ > 0.5 were pruned into a single independent SNP using the PLINK command –indep-pairwise 25 5 0.5. Allele frequencies (AF) between different populations were compared using the Pearson Chi2 test, with a *p*-value < 0.05 considered statistically significant.

## Results

A total of 74 SNPs were identified from the literature. These SNPs are located in 10 genes: *ICAM3, IFN-γ, CCL2, CCL5, AHSG, MBL, Furin, TMPRSS2, IL4*, and *CD209* promoter. Table 1 summarizes the list of all SARS-CoV-1 related SNPs reported by previous GWAS (*n* = 11) and their potential effect during infection.

**TABLE 1:** Common single nucleotide polymorphisms in host genes related to SARS-CoV-1 infection.

**Gene**	**CHR**	**Position**	**SNP ID**	**Risk allele**	**Alternative Allele**	**Consequence**	**GWAS phenotype/Disease**	**Study population**	**Effect**	**References**
***CCL2***	17	32579788	rs1024611	G	A	Upstream gene variant	SARS-CoV	Chinese	Increased transcriptional activity of *CCL2*- Higher susceptibility to SARS-CoV-1	Tu et al., 2015
***MBL***	10	54531235	rs1800450	T	C	Missense variant	SARS-CoV	Chinese	Lower *MBL* expression- Higher susceptibility to SARS-CoV-1	Tu et al., 2015
***RANTES (CCL5)***	17	34207405	rs2280788	C	G	Upstream gene variant	SARS-CoV	Chinese	Higher expression of *CCL5* Higher SARS-CoV-1 severity	Ng et al., 2007
***AHSG***	3	186330088	rs2248690	T	A	Upstream gene variant	SARS-CoV	Chinese	Higher production of *AHSG* Lower SARS-CoV-1 severity	Zhu et al., 2011
***ICAM3***	19	10446568	rs2304237	C	T	Missense variant	SARS-CoV	Chinese	Higher *ICAM3* expression- Higher LDH levels- Higher SARS-CoV-1 severity	Chan et al., 2007
***IFN*-γ**	12	68552522	rs2430561	A	T	Intron variant	SARS-CoV	Chinese	Decreased *IFN*-γ production Higher SARS-CoV-1 severity	Chong et al., 2006
***CD209 promoter***	19	7812733	rs4804803	A	G	Upstream gene variant	SARS-CoV	Chinese	Altered *CD209* gene expression Higher LDH levels Poor SARS-CoV-1 prognosis	Chan et al., 2010
***IL4***	5	132009710	rs2070874	T	C	5′ UTR variant	SARS and other respiratory infections	NA	Altered IL4 production- Higher susceptibility to SARS-CoV-1	Patarčić et al., 2015
***TMPRSS2***	21	42841988	rs2070788	G	A	Intron variant	Influenza	Chinese	Higher *TMPRSS2* expression Higher susceptibility to influenza	Cheng et al., 2015
		42858367	rs383510	T	C	Intron variant	Influenza	Chinese	Higher *TMPRSS2* expression Higher susceptibility to influenza	
***Furin***	15	91411656	rs4932178	T	C	Upstream gene variant	HBV	Chinese	Higher *Furin* expression Higher susceptibility to HBV	Lei et al., 2009

*NA, Data not available; CHR, chromosome number; 1000G, 1000Genome project data; QAT, Qatari; AFR, African; AMR, American; EAS, Eastern Asian; EUR, European; SAS, South Asian.*

**FIGURE 1:**
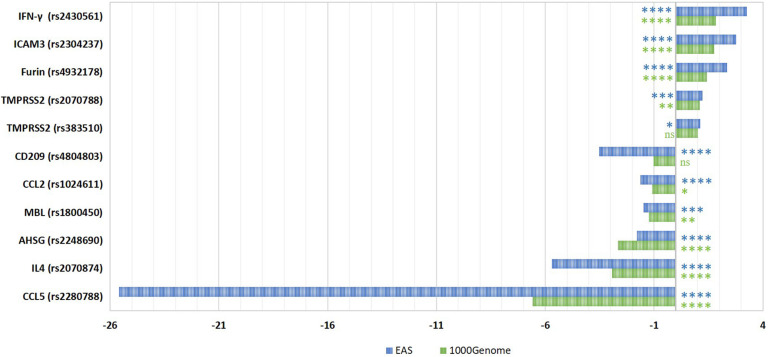
Fold differences in the risk allelic frequencies of SARS-CoV-1-related SNPs among Qatari population in comparison to 1000Genome and Eastern Asian populations. The fold change in AF was calculated for each SNP. The significance of AF difference between each two groups was calculated using Pearson Chi2 test. *P*-value less than 0.05 is flagged with one star (*). *P*-value less than 0.01 is flagged with two stars (**). *P*-value less than 0.001 is flagged with three stars (***). *P*-value less than 0.0001 is flagged with four stars (****). Ns, not significant. 1000G, 1000Genome project data; EAS, Eastern Asian.

We first compared the frequencies of SARS-CoV-1 related SNPs in Qatari population genomes (*n* = 6047) to the global AFs from the 1000Genome dataset (The International Genome Sample Resource [IGSR], 2015). In addition, we calculated the fold difference in the AF in comparison to the Eastern Asian (EAS) population specifically (*n* = 504), since all these susceptibility SNPs were originally identified in the Chinese population (Figure 1). Analysis of Qatari genomes revealed that the Qatari population has significantly lower frequency of the risk alleles in each of: *CCL2*, *MBL*, *CCL5*, *AHSG*, and *IL4* (0.336, 0.099, 0.004, 0.09, and 0.137, respectively), compared to the 1000Genome (0.367, 0.122, 0.025, 0.24, and 0.401, respectively), as well to the EAS population (0.547, 0.148, 0.095, 0.161, and 0.779, respectively). Remarkably, the most significant difference was observed in rs2280788 that is located in the *CCL5* gene. The frequency of the risk allele (C) among Qatari population was 6.6-fold lower than the 1000Genomes (0.372 vs. 2.4%), and 25.6-fold lower than that of the EAS population (9.52%). Second in line was rs2070874 in *IL4* gene, where the frequency of the risk variant (T) among Qataris showed around 3-fold decrease (13.7%) compared to the global AF (40.1%), and a 5.7-fold decrease compared to EAS population (77.9%). Additionally, the *AHSG* risk variant (rs2248690, risk allele = T), was less frequently detected among Qataris (AF = 0.09), with a 2.7-fold decrease compared to the EAS population (AF = 0.16). However, although SNPs in *CCL2* and *MBL* genes were significantly different among the Qatari population, the fold difference was marginal (1 to 2-fold change). On the other hand, a higher frequency of the risk alleles located within *IFN*-γ, *ICAM3*, *Furin*, and *TMPRSS2* was observed in the Qatari population (0.517, 0.314, 0.376, and 0.433, respectively), in comparison to 1000Genomes (0.28, 0.18, 0.264, and 0.396, respectively), and to the EAS population (0.159, 0.113, 0.16, and 0.36, respectively). Notably, Qatari genomes showed 3.3- and 2.8-fold increase in the AF of *IFN*-γ and *ICAM3* SNPs compared to the EAS population. The AF of *CD209* variant among Qataris was comparable to that of 1000Genomes; however, it showed a 3.5-fold decrease in comparison to the EAS population.

**FIGURE 2:**
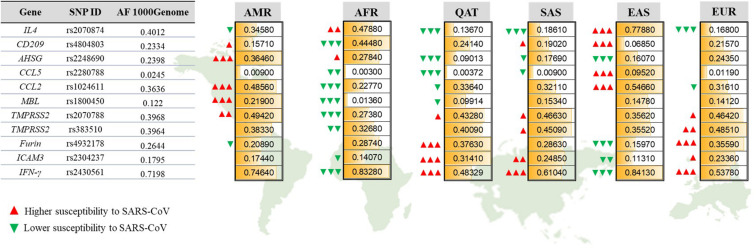
Allelic frequencies of SARS-CoV-1-associated genetic variants among different populations. Representation of population-specific differences in the AFs of 11 SARS-CoV related SNPs located within 10 genes, in comparison to the overall AF from 1000Genome. AMR, American; AFR, African; QAT, Qatari; SAS, South Asian; EAS, Eastern Asian; EUR, European. Green and red arrows indicate the effect of each AF on increasing (red) or decreasing (green) susceptibility to SARS-CoV-1. The number of arrows represents the significance level of difference in AF for each population compared to the 1000Genome. *P*-value less than 0.05 is flagged with one arrow (Δ). *P*-value less than 0.01 is flagged with two arrows (ΔΔ). *P*-value less than 0.0001 is flagged with three arrows (ΔΔΔ).

We further explored the inter-population variations for each SNP to identify significant differences (Figure 2). The *CCL5* risk allele (C, rs2280788) was considerably more common among people from EAS (0.952), reaching to a 32-fold increase compared to the African and Qatari populations that exhibited the lowest frequency of this risk allele (0.003 of each population). Similarly, the percentage of individuals harboring the risk allele (T) in *IL4* (rs2070874) was the highest among EAS population (77.9%) and the lowest among European population (16.8%). Likewise, the risk allele (A) of SNP rs4804803 in *CD209* promoter, as well as *CCL2* risk variant (G, rs1024611) were significantly higher among EASs compared to all other populations (93.15%, and 54.66%, respectively), while the Africans had the lowest percentages of both mutations (55.52% and 22.77%, respectively). Africans showed an extremely lower AF of *MBL* risk variant (T, rs1800450, AF = 0.013), which is 9-folds less than the global AF (0.122), while Americans presented the highest AF (0.219). Likewise, the frequency of the risk allele (G) of *TMPRSS2* rs2070788 was the lowest among Africans (0.2738), and the highest in Americans (0.4942). However, the other SNP that also affects *TMPRSS2* expression (rs383510) was more frequent among European population (0.4851), while African population also exhibited the lowest frequency (0.3268). The distribution of the T risk allele of *AHSG* gene was similarly high among Africans and Americans (0.2784 and 0.3646, respectively), but less detected among Qatari population (9%). Qatari individuals, on the other hand, showed the highest AF in risk variants of *Furin* (T, rs4932178, 0.3763), *ICAM3* (C, rs2304237, 0.3141), and *IFN*-γ (A, rs2430561, 0.5167) in comparison to all other populations.

Lastly, for the same set of genes, we investigated the genetic variations that were already annotated as eQTLs in the whole blood, which might affect disease susceptibility or severity. We also used QGP dataset and as well as data deposited in 1000Genome for the analysis. Only four out of ten genes had significant associations in the eQTL databases: *CCL5*, *CD209*, *ICAM3*, and *MBL*, where 63 eQTLs were located (Supplementary Table S1). Of these, only ten variants were found to be independent SNPs and were further investigated. Table 2 demonstrates the eQTLs included in the analysis and their suggested effect on gene expression. Our analysis showed that the frequency of almost all these eQTL variants differs considerably between populations. Mainly, African ancestry has up to 4-fold decrease in the frequency of eQTLs in *CD209* (rs10518270), and *ICAM3* (rs3181049 and rs3176767). The European population, on the other hand, had the lowest frequency of rs4239252 in *CCL5* (2.4-fold difference). Of note, other eQTLs appear to vary between populations, yet, with a lower fold difference (Table 3).

**TABLE 2:** Independent eQTL variants in host genes related to SARS-CoV-1 infection.

**Gene**	**CHR**	**Position**	**SNP ID**	**Minor/Major allele**	**Risk allele**	**Consequence**	**SNP effect on gene expression**	***P*-value**
***CCL5***	17	34163565	rs4239252	A/G	A	Intron variant	Downregulation	2 × 10^–7^
***CD209***	19	7781435	rs10518270	G/A	G	Intergenic variant	Downregulation	2.9 × 10^–4^
		7785713	rs2335525	G/A	G	Regulatory region variant	Downregulation	8.8 × 10^–4^
		7807610	rs4804802	A/G	G	Downstream gene variant	Downregulation	0.1
***ICAM3***	19	10441117	rs3181049	A/G	A	Downstream gene variant	Downregulation	1.8 × 10^–3^
		10449751	rs3176767	G/T	G	Intron variant	Downregulation	6 × 10^–4^
		10457917	rs4611572	C/G	G	Downstream gene variant	Downregulation	3.7 × 10^–8^
		10446734	rs281413	A/G	A	Intron variant	NA	–
		10449392	rs2304240	A/G	G	Synonymous variant	Downregulation	1.6 × 10^–17^
***MBL***	10	54531685	rs7096206	G/C	C	Upstream gene variant	NA	–

*NA, Data not available; CHR, chromosome number; 1000G, 1000Genome project data; QAT, Qatari; AFR, African; AMR, American; EAS, Eastern Asian; EUR, European; SAS, South Asian.*

**TABLE 3:** Allelic frequency of eQTL in genes related to SARS-CoV-1 infection.

**Gene**	**SNP ID**	**Minor/Major allele**	**Risk allele**	**Minor allele frequency**						
				**1000G**	**QAT**	**AFR**	**AMR**	**EAS**	**EUR**	**SAS**
***CCL5***	rs4239252	A/G	A	0.39600	0.22780	0.60670	0.28820	0.45630	0.16200	0.36610
***CD209***	rs10518270	G/A	G	0.18910	0.24860	0.04390	0.09510	0.31150	0.14020	0.37630
	rs2335525	G/A	G	0.40590	0.41350	0.68000	0.18590	0.32540	0.26240	0.42230
	rs4804802	A/G	G	0.21630	0.13970	0.33360	0.09650	0.31450	0.11430	0.14620
***ICAM3***	rs3181049	A/G	A	0.12520	0.28359	0.03780	0.15850	0.08230	0.21470	0.17180
	rs3176767	G/T	G	0.17930	0.31690	0.13920	0.17440	0.11410	0.23560	0.24640
	rs4611572	C/G	G	0.47700	0.48540	0.62030	0.51440	0.34230	0.42150	0.45300
	rs281413	A/G	A	0.16830	0.12980	0.23150	0.14410	0.08530	0.19580	0.15750
	rs2304240	A/G	G	0.17170	0.11998	0.06280	0.33290	0.17460	0.17690	0.19630
***MBL***	rs7096206	G/C	G	0.19550	0.28450	0.15360	0.13110	0.18550	0.22070	0.28220

*1000G, 1000Genome project data; QAT, Qatari; AFR, African; AMR, American; EAS, Eastern Asian; EUR, European; SAS, South Asian.*

## Discussion

Since the start of the current SARS-CoV-2 pandemic, scientists have been puzzling over the factors underlying the inter-individual and inter-population differences in disease outcomes. The resulting clinical manifestation of COVID-19 varied enormously, ranging from mild/asymptomatic illness in 80% of patients to a severe respiratory syndrome in 20%, which further progresses to critical illness requiring ventilation in 5% (CDC, 2020). In addition, the mortality rates were interestingly different between countries. As of August 5, 2020, the highest mortality rate was seen in European countries such as in the United Kingdom, Italy, and France, reaching 15% (Statistica, 2020). On the contrary, the lowest rate of death from COVID-19 was reported from several Western and South Asian countries such as Singapore (0.06%), Qatar (0.1%), and Bahrain (0.27%) and African countries such as Rwanda (0.24%) and Uganda (0.42%) (Statistica, 2020). These figures are affected by several factors, including the testing capacity in each country, the age and health status of individuals, the efficiency of the health system, and the possible circulation of different viral strains. However, population genetics can also be a key factor. The earlier SARS-CoV-1 outbreak in 2003 had shed some light on the host genetic contribution in disease manifestations and outcomes. A number of GWASs have identified genetic markers with associations with SARS-CoV-1, yet, all were conducted on Chinese population, where SARS-CoV-1 originated and mainly circulated. Nevertheless, to the best of our knowledge, the prevalence and frequency of these variants have never analyzed in comparison to other populations, including the Middle East and North Africa (MENA) population. Accordingly, in this report, we performed a comparative analysis on a set of genetic polymorphisms that could have a potential effect on SARS-CoV-2 susceptibility or severity, utilizing the datasets from Qatar Genome Programme (QGP), as well as the 1000Genomes.

Initially, we looked at the overall burden of risk variants associated with SARS-CoV-1 in the Qatari population. These variants are mostly located in genes involved in viral entry (*TMPRSS2* and *Furin*), cytokine production (*IFN*-γ and *IL4*), and immune responses (*ICAM3*, *CCL2*, *CCL5*, *AHSG*, *MBL*, and *CD209*). Our analysis showed a remarkable decrease in the risk allelic frequencies of SNPs linked to SARS severity in the Qatari population, such as *IL4*, *AHSG*, *CCL5*, and *CCL2* variants. However, the Qatari population had a significantly higher frequency of SNPs related to increased disease susceptibility, including *TMPRSS2*, *Furin*, and *IFN*-γ variants. The only genetic polymorphism associated with SARS-CoV-1 severity and found at a higher rate among Qataris is the *ICAM3* gene polymorphism (rs2304237). This variant was previously reported to upregulate *ICAM3* gene expression and increase SARS-CoV-1 severity. Nonetheless, two eQTLs in the same gene (rs3181049 and rs3176767), which are responsible for downregulating *ICAM3* expression and potentially decreasing SARS-CoV-1 severity, were detected at the highest rate among Qataris. These findings align with the current situation in Qatar, where high rates of SARS-CoV-2 infections are reported despite the low severity (ICU cases = 1%), and mortality rates (0.1%) (Ministry of Public Health [MOPH], 2020). Although this data mostly represent the disease spread among expatriates who represent 85% of Qatar population, Qatari citizens also have extremely low fatality rates (0.07%, personal communication, June 2020).

Investigating the differences in the distribution of SARS-CoV-1 associated SNPs and eQTLs in different populations showed great diversity. The fold difference in AFs between populations reached up to 32-fold change as observed in rs2280788 *(CCL5 gene)* which was found in 9.5% of EAS population compared to 0.3% only among Africans and Qataris. Similarly, rs1800450 in the *MBL* gene showed a high variability between populations (16-fold difference). This variant was found in 22% of Americans compared to only 1.36% of Africans. This data highlights again the importance of explaining infections spread and pathogenesis in the light of population genetics. Noticeably, the European population showed an opposite fold direction in the allelic frequency of most SNPs compared to the African and EAS propulsions. In other words, all risk variants, which were more commonly detected in Europeans (*TMPRSS2*, *Furin*, *ICAM3*, and *IFN*-γ), were significantly lower among Africans and EASs. In fact, compared to all other populations, African descent seems to carry substantially lower frequencies of the risk alleles in most SNPs (8 out of 11 SNPs). Previous population genetics studies pointed out the great differences in the amplitude of the immune response between Africans and Europeans, especially in genes related to inflammatory and antiviral responses (Quach et al., 2016). In agreement with our findings, a recent study also showed that African have a genetic predisposition for lower expression levels of both *ACE2* and *TMPRSS2* genes, which are key viral entry genes in SARS-CoV-2 infection (Ortiz-Fernández and Sawalha, 2020). Collectively, this could explain the population differences in COVID-19 infection and fatality rates. Africa is still considered an outlier in terms of COVID-19 spread. The number of cases in Africa (as of August 5, 2020) is the lowest (981,593 positive cases), compared to other continents (3–5 million positive cases) (Worldometers, 2020). Additionally, despite their fragile health system, Africans are still reporting a relatively low mortality rate (2.2%), compared to the global death rate, which is 3.7% (Worldometers, 2020). Nonetheless, it is worth mentioning that the limited testing capacity in African countries could largely underestimate the actual burden. To the contrary, it has been reported that the death rate from COVID-19 is 6-fold higher among African Americans counties compared to predominantly white counties (Yancy, 2020). Whether this observation is solely related to socioeconomic and cultural factors or also affected by genetic factors, require further investigation.

Although SARS-CoV-1 and SARS-CoV-2 were originally identified in EAS (China), our analysis revealed that only three susceptibility loci were significantly higher among the EASs (*IL4*, *CD209* promoter, and *CCL2*). This could be attributed to the limited set of variants included in our analysis and does not exclude the possibility of the EAS population being differently vulnerable to coronaviruses. A recent study reported that EAS individuals have higher allele frequencies in the eQTL variants associated with augmented *ACE2* expression in tissues, suggesting a possible different susceptibility or response to SARS-CoV-2 (Cao et al., 2020). Nonetheless, additional well-designed studies, larger in size and scope, are needed to better characterize the population differences in SARS-CoV2 susceptibility and disease outcomes.

One of the main clinical features of severe COVID-19 is the exacerbated inflammatory response. Both SARS-CoV-1 and SARS-CoV-2 infections are known to induce a massive over-release of cytokines, which contributes to infection pathogenesis and severity. SARS-CoV-infected cells produce high levels of chemokines, including CCL2, and CCL5, and proinflammatory cytokines such as IFN-γ (Chang et al., 2020). We found that variants, which increase the expression of these molecules specifically, are distributed differently among populations, and consequently, will differ between individuals. These variants could be useful as prognostic markers to stratify patients and identify high-risk individuals.

In conclusion, we highlight here the population-dependent variations in genes potentially influencing SARS-CoV-2 infection. Results from this work emphasize the importance of understanding the interplay between host genetic factors and response to infections, which could have important implications on public health infections control and therapeutics. Importantly, results presented here provide preliminary insights that necessitate functional validation in future studies.

## Data Availability Statement

All datasets presented in this study are included in the article/Supplementary Material.

## Ethics Statement

The studies involving human participants were reviewed and approved by the Qatar University Institutional Review Board (IRB), approval no. QU-IRB 1287-EA/20, and Qatar Biobank IRB, approval no. E-2020-QBB-RES-ACC-0184-0110. Written informed consent for participation was not required for this study in accordance with the national legislation and the institutional requirements.

## Author Contributions

HY developed the concept. OA supervised the data curation and analysis. MS and YA-S performed the analysis and wrote the first draft of the manuscript. HY and OA revised the manuscript. All authors contributed to the article and approved the submitted version.

## Conflict of Interest

The authors declare that the research was conducted in the absence of any commercial or financial relationships that could be construed as a potential conflict of interest.

## Footnotes

1 http://www.exsnp.org/

2 http://eqtl.uchicago.edu/Home.html

3 https://gtexportal.org/home/
