# Supplementary material for: Host Genetic Variants Potentially Associated With SARS-CoV-2: A Multi-Population Analysis
Source: Front Genet. 2020 Oct 2;11:578523. doi: 10.3389/fgene.2020.578523 (PMC7567011; doi:10.3389/fgene.2020.578523)
Supplement: Supplementary file 1 [file Table_1.DOCX]

# Supplementary Material

**Table S1. Full list of eQTL variants in host genes related to SARS-CoV-1 infection**

| **Gene** | **SNP ID** |  | **CHR** | **Minor allele** | **Major Allele** | **Minor Allele Frequency** | | | | | | |
| --- | --- | --- | --- | --- | --- | --- | --- | --- | --- | --- | --- | --- |
|  |  |  |  |  |  | **1000G** | **QAT** | **AFR** | **AMR** | **EAS** | **EUR** | **SAS** |
| ***CCL5*** | rs4239252 |  | 17 | A | G | 0.39600 | 0.22780 | 0.60670 | 0.28820 | 0.45630 | 0.16200 | 0.36610 |
|  | rs4796123 |  | 17 | C | T | 0.17910 | 0.14300 | 0.23980 | 0.19600 | 0.22920 | 0.11130 | 0.10330 |
|  | rs4251719 |  | 17 | A | G | 0.18310 | 0.14500 | 0.18680 | 0.19880 | 0.30750 | 0.11330 | 0.11040 |
|  | rs4251769 |  | 17 | T | C | 0.18410 | 0.14500 | 0.19210 | 0.19880 | 0.30650 | 0.11330 | 0.10940 |
|  | rs2280789 |  | 17 | G | A | 0.18530 | 0.14260 | 0.19210 | 0.20030 | 0.30750 | 0.11330 | 0.11350 |
|  | rs4251737 |  | 17 | A | C | 0.18130 | 0.13440 | 0.18680 | 0.19310 | 0.30360 | 0.11330 | 0.10940 |
|  | rs9889874 |  | 17 | T | G | 0.17870 | 0.13213 | 0.17870 | 0.19500 | 0.30750 | 0.11030 | 0.10900 |
|  | rs2306630 |  | 17 | A | G | 0.18350 | 0.13280 | 0.19140 | 0.19880 | 0.30750 | 0.11130 | 0.10840 |
|  | rs9303692 |  | 17 | A | G | 0.18390 | 0.13290 | 0.19210 | 0.19880 | 0.30750 | 0.11130 | 0.10940 |
|  | rs11653282 |  | 17 | T | C | 0.18710 | 0.13410 | 0.21180 | 0.19740 | 0.30560 | 0.10740 | 0.10630 |
|  | rs11868785 |  | 17 | G | A | 0.18830 | 0.13250 | 0.18910 | 0.19880 | 0.33130 | 0.11330 | 0.10940 |
|  | rs3826404 |  | 17 | G | T | 0.28210 | 0.18300 | 0.36160 | 0.21330 | 0.31550 | 0.16500 | 0.30980 |
|  | rs2526327 |  | 17 | A | G | 0.27400 | 0.17700 | 0.44400 | 0.20170 | 0.21030 | 0.14510 | 0.29350 |
|  | rs3817655 |  | 17 | T | A | 0.28160 | 0.17380 | 0.45390 | 0.22050 | 0.32140 | 0.15900 | 0.17690 |
|  | rs4796105 |  | 17 | C | A | 0.28550 | 0.17500 | 0.39330 | 0.21610 | 0.27380 | 0.16000 | 0.33030 |
|  | rs7211393 |  | 17 | T | C | 0.30850 | 0.18880 | 0.45920 | 0.22480 | 0.31550 | 0.16100 | 0.30880 |
|  | rs4251703 |  | 17 | C | T | 0.31510 | 0.19080 | 0.46600 | 0.22050 | 0.35120 | 0.15710 | 0.30370 |
|  | rs4079183 |  | 17 | G | A | 0.30890 | 0.18590 | 0.45840 | 0.22480 | 0.32340 | 0.16000 | 0.30470 |
|  | rs112650587 |  | 17 | CAT | C | 0.30850 | 0.18555 | 0.45840 | 0.22330 | 0.32340 | 0.16000 | 0.30370 |
|  | rs4795090 |  | 17 | G | A | 0.31550 | 0.18940 | 0.46670 | 0.22050 | 0.35020 | 0.15900 | 0.30370 |
|  | rs11650668 |  | 17 | A | T | 0.30830 | 0.18408 | 0.45760 | 0.22480 | 0.32240 | 0.16000 | 0.30370 |
|  | rs4251724 |  | 17 | A | T | 0.31370 | 0.18600 | 0.45840 | 0.22620 | 0.34920 | 0.15610 | 0.30570 |
|  | rs4251725 |  | 17 | TA | T | 0.31370 | 0.18590 | 0.45840 | 0.22620 | 0.34920 | 0.15610 | 0.30570 |
|  | rs8069014 |  | 17 | A | G | 0.29710 | 0.17390 | 0.45390 | 0.17720 | 0.31050 | 0.15310 | 0.30470 |
|  | rs8066406 |  | 17 | T | C | 0.38820 | 0.22670 | 0.57410 | 0.28390 | 0.45440 | 0.15810 | 0.37930 |
|  | rs55858632 |  | 17 | G | A | 0.32530 | 0.18800 | 0.50760 | 0.23490 | 0.31650 | 0.16600 | 0.31600 |
|  | rs28695841 |  | 17 | C | G | 0.33190 | 0.19002 | 0.56580 | 0.22770 | 0.30060 | 0.15710 | 0.30160 |
|  | rs4796128 |  | 17 | A | G | 0.30430 | 0.17320 | 0.45010 | 0.22480 | 0.31450 | 0.15900 | 0.30270 |
|  | rs7215845 |  | 17 | T | G | 0.30550 | 0.17380 | 0.45310 | 0.22190 | 0.31550 | 0.15900 | 0.30570 |
|  | rs4251787 |  | 17 | A | G | 0.30790 | 0.17490 | 0.44630 | 0.22190 | 0.32240 | 0.15900 | 0.32000 |
|  | rs6505496 |  | 17 | G | A | 0.31830 | 0.18070 | 0.47580 | 0.22190 | 0.35320 | 0.15710 | 0.30370 |
|  | rs35283303 |  | 17 | A | AG | 0.30410 | 0.17257 | 0.44630 | 0.22190 | 0.32240 | 0.15810 | 0.30160 |
|  | rs4795087 |  | 17 | G | C | 0.33650 | 0.19050 | 0.53860 | 0.23900 | 0.32140 | 0.16600 | 0.32300 |
|  | rs2291299 |  | 17 | C | T | 0.30830 | 0.17410 | 0.45840 | 0.22480 | 0.32240 | 0.15810 | 0.30470 |
|  | rs3760327 |  | 17 | C | G | 0.30850 | 0.17420 | 0.54160 | 0.23300 | 0.32240 | 0.16000 | 0.30500 |
|  | rs4796116 |  | 17 | C | G | 0.30850 | 0.17420 | 0.45840 | 0.22480 | 0.32240 | 0.16000 | 0.30370 |
|  | rs78655848 |  | 17 | A | G | 0.30790 | 0.17370 | 0.45920 | 0.22330 | 0.32240 | 0.15810 | 0.30270 |
|  | rs8080959 |  | 17 | A | G | 0.30810 | 0.17360 | 0.45840 | 0.22480 | 0.31940 | 0.16000 | 0.30470 |
|  | rs9908928 |  | 17 | G | A | 0.30790 | 0.17330 | 0.45760 | 0.22330 | 0.32240 | 0.15810 | 0.30470 |
|  | rs28595279 |  | 17 | T | G | 0.30850 | 0.17340 | 0.45840 | 0.22330 | 0.32240 | 0.16000 | 0.30470 |
|  | rs4251749 |  | 17 | T | C | 0.30890 | 0.17340 | 0.45840 | 0.22480 | 0.32340 | 0.16000 | 0.30470 |
|  | rs4325615 |  | 17 | A | G | 0.30850 | 0.17310 | 0.45840 | 0.22330 | 0.32240 | 0.16000 | 0.30470 |
|  | rs35517765 |  | 17 | C | T | 0.33550 | 0.18380 | 0.52190 | 0.23630 | 0.35320 | 0.16500 | 0.31080 |
|  | rs1994182 |  | 17 | G | C | 0.43570 | 0.21880 | 0.65050 | 0.29540 | 0.57240 | 0.16700 | 0.38040 |
|  | rs4251791 |  | NA | NA | NA | 0.31130 | NA | 0.45840 | 0.22330 | 0.32240 | 0.15900 | 0.32000 |
| ***CD209*** | rs10518270 |  | 19 | G | A | 0.18910 | 0.24860 | 0.04390 | 0.09510 | 0.31150 | 0.14020 | **0.37630** |
|  | rs2335525 |  | 19 | G | A | 0.40590 | 0.41350 | **0.68000** | 0.18590 | 0.32540 | 0.26240 | 0.42230 |
|  | rs4804802 |  | 19 | A | G | 0.21630 | 0.13970 | **0.33360** | 0.09650 | 0.31450 | 0.11430 | 0.14620 |
|  | rs11260025 |  | 19 | C | T | 0.18810 | 0.24860 | 0.04010 | 0.09510 | 0.31150 | 0.14020 | 0.37630 |
|  | **rs8105572** |  | 19 | T | C | 0.14140 | 0.12094 | 0.23900 | 0.08500 | 0.06550 | 0.15610 | 0.11250 |
| ***ICAM3*** | rs3181049 |  | 19 | A | G | 0.12520 | 0.28359 | 0.03780 | 0.15850 | 0.08230 | 0.21470 | 0.17180 |
|  | rs3176767 |  | 19 | G | T | 0.17930 | 0.31690 | 0.13920 | 0.17440 | 0.11410 | 0.23560 | 0.24640 |
|  | rs4611572 |  | 19 | C | G | 0.47700 | 0.48540 | 0.62030 | 0.51440 | 0.34230 | 0.42150 | 0.45300 |
|  | rs281413 |  | 19 | A | G | 0.16830 | 0.12980 | 0.23150 | 0.14410 | 0.08530 | 0.19580 | 0.15750 |
|  | rs2304240 |  | 19 | A | G | 0.17170 | 0.11998 | 0.06280 | 0.33290 | 0.17460 | 0.17690 | 0.19630 |
|  | rs3176766 |  | 19 | A | G | 0.17910 | 0.31690 | 0.13920 | 0.17440 | 0.11410 | 0.23460 | 0.24640 |
|  | rs3176768 |  | 19 | T | C | 0.17930 | 0.31690 | 0.13920 | 0.17440 | 0.11410 | 0.23560 | 0.24640 |
|  | rs1058154 |  | 19 | A | C | 0.16890 | 0.31400 | 0.13690 | 0.17580 | 0.07540 | 0.22960 | 0.24130 |
|  | rs7258015 |  | 19 | C | T | 0.17890 | 0.31660 | 0.13920 | 0.17440 | 0.11410 | 0.23560 | 0.24440 |
|  | rs7257871 |  | 19 | C | T | 0.17930 | 0.31670 | 0.13990 | 0.17440 | 0.11410 | 0.23560 | 0.24540 |
|  | rs3745264 |  | 19 | A | C | 0.16510 | 0.10960 | 0.05750 | 0.29680 | 0.17760 | 0.15710 | 0.21270 |
| ***MBL2*** | rs7096206 |  | 10 | G | C | 0.19550 | 0.28450 | 0.15360 | 0.13110 | 0.18550 | 0.22070 | 0.28220 |

1000G: 1000Genome project data, QAT: Qatari, AFR: African, AMR: American, EAS: Eastern Asian, EUR: European, SAS: South Asian.
